# 

**Published:** 1833-04

**Authors:** 


### MONTHLY LIST OF MEDICAL BOOKS.

[*Medical Works cannot be entered on this List unless a copy be sent for the purpose, the titles of Books having frequently been sent to us as published, which have not appeared for weeks, or even months, after.*]

The Dissector. By R. D. Forster, Surgeon. Part I. Folio. Six Plates, coloured.—Burgess and Hill, London.

The Demonstrator; being an Explanation of the Dissector of the Human Body. By R. D. Forster, Surgeon. In sixteen Parts. Part I.—8vo. pp. 50. Burgess and Hill, London.

This work deserves encouragement. It will prove extremely useful to students, by recalling to their minds any anatomical facts which may have escaped their recollection; and it is admirably adapted to enable practitioners, who cannot attend the dissecting room, to keep up their anatomical knowledge at little expense of time or labour.

A Treatise on the Physiology and Diseases of the Eye: containing a new Mode of curing Cataract without an Operation; Experiments and Observations on Vision, also on the Inflection, Reflection, and Colours of Light. Together with Remarks on the Preservation of Sight, and on Spectacles, Reading Glasses, &c. By John Harrison Curtis, Esq. Oculist; Aurist to his Majesty, &c.—8vo. pp. 222. Longman and Co., London.

A Treatise on Indigestion and its Consequences, called Nervous and Bilious Complaints; with Observations on the Organic Diseases in which they sometimes terminate. By A. P. W. Philip, m.d., f.r.s. l. and e., &c. *Seventh Edition.*—8vo. pp. 231. Renshaw and Rush, London.

Part III., March 1833. Principles and Illustrations of Morbid Anatomy; adapted to the Elements of M. Andral, and to the Cyclopædia of Practical Medicine (with which it will correspond in size). Being a complete Series of coloured Lithographic Drawings, from Originals by the Author; with Descriptions and summary Allusions to Cases, Symptoms, Treatment, &c. Designed to constitute an Appendix to Works on the Practice of Physic, and to facilitate the Study of Morbid Anatomy in connexion with Symptoms. By J. Hope, m.d., f.r.s., Physician to the St. Marylebone Infirmary, &c.—Whittaker and Co., London.

Outlines of the Course of Lectures on Military Surgery, delivered in the University of Edinburgh, by Sir George Ballingall, m.d., f.r.s.e., Regius Professor of Military Surgery, &c.—8vo. pp. 589. Black, Edinburgh; Longman, London.

On the Mind, and Nature of Human Knowledge; being the Substance of a Paper read to the Literary Society, Bromley House, by George Cox, m.d.—8vo. pp. 67. Simpkin and Marshall, London.

A Practical Appeal to the Public, through a Series of Letters, in Defence of the New System of Physic by the illustrious Hahnemann. Letter the First. By John Borthwick Gilchrist, ll.d. &c.—8vo. pp. 100. Parbury, Allen, and Co., London.

No. I. Hortus Medicus; or, Figures and Descriptions of the more important Plants used in Medicine, or possessed of Poisonous Qualities; with their Medical Properties, Chemical Analysis, &c. &c. By George Graves, Fellow of the Linnæan Society, Editor of the new Edition of Curtis's Flora Londinensis, Author of a Monograph on the British Grasses, &c.; and John Davie Morries, m.d., Member of the Medico-Chirurgical, Royal Medical, and Plinian Societies of Edinburgh, &c.—4to. pp. 32; ten Plates. Black, Edinburgh; Longman, London.

### APOTHECARIES' HALL.

**Names of Gentlemen** to each of whom the Court of Examiners have granted Certificates:

FEB. 28.

William Day, of Hull
Charles Dilke, Appleby, Leicestershire
Frederiak Disting Tothill, Exeter
Edward Joseph Weston, Kennington.

MARCH 7.

Peter Brown, of Thackley, Yorkshire
Thomas Bradley, London
Thomas Lambert Hinton, Daglingworth
Edwin Moss, Cheltenham
Robert Nixon, Wigton
Alfred Pett, London
John Phillips, Newmoat, Pembroke
John Simpson Rutter, Lichfield
Robert Holden Stone, Brasted, Kent
William Thomas, Minichinhampton.

MARCH 14.

William Carfrae, of Edinburgh
Douglas Dutton, London
Henry Whitworth Kerr, Manchester
Edward William Pollard, Brompton
James Yates Rooker, Darlaston
Thomas Sweeny, Market Street
John Wightman
Henry Wilson, Runcorn
Michael William Kansby, Abergavenny

MARCH 21.

John Grayling, of Canterbury
George Dowler Hastlewood, Bsrmingham
Thomas Goldson Irving, Portsmouth
Thomas Philbrick, Witham, Essex
Alfred Prentice, Essex.

### TO CORRESPONDENTS.

Mr. Ballard *will excuse us for not publishing his remarks on the subject of* "Medical Electricity:" *we admit their ingenuity, but we do not think the facts are as yet sufficiently substantiated to admit, with propriety, of any hypothesis upon the subject. We shall always be happy to hear from* Mr. B.

J. G.—*A paper will soon be published in this Journal upon the subject, in which references will be given to various foreign and English authors who have written on it.*

